# Influence of Harvesting Stage on Phytochemical Composition, Antioxidant, and Antidiabetic Activity of Immature *Ceratonia siliqua* L. Pulp from Béni Mellal-Khénifra Region, Morocco: In Silico, In Vitro, and In Vivo Approaches

**DOI:** 10.3390/cimb46100653

**Published:** 2024-09-29

**Authors:** Salah Laaraj, Hanane Choubbane, Amal Elrherabi, Aziz Tikent, Ayoub Farihi, Meriem Laaroussi, Mohamed Bouhrim, Abdelaaty A. Shahat, Younes Noutfia, Rashed N. Herqash, Fatiha Chigr, Souad Salmaoui, Kaoutar Elfazazi

**Affiliations:** 1 Agri-Food Technology and Quality Laboratory, Regional Centre of Agricultural Research of Tadla, National Institute of Agricultural Research (INRA), Avenue Ennasr, Bp 415 Rabat principal, Rabat 10090, Morocco; 2 Environmental, Ecological and Agro-Industrial Engineering Laboratory, LGEEAI, Faculty of Science and Technology (FST), Sultan Moulay Slimane University (USMS), Beni Mellal 23000, Morocco; souadsalmaoui@yahoo.fr; 3 Laboratory of Sustainable Development and Health, Faculty of Science and Technology Guéliz (FSTG), University Cadi Ayyad of Marrakech, Marrakech 40000, Morocco; hanane.choubbane@edu.uca.ac.ma; 4 Laboratory of Bioresources, Biotechnology, Ethnopharmacology and Health, Faculty of Sciences, Mohammed First University, Bp 717, Oujda 60000, Morocco; amal.rhe96@gmail.com; 5 Laboratoire d’Amélioration des Productions Agricoles, Biotechnologie & Environnement (LAPABE), Faculté des Sciences, Université Mohammed Premier, Bp 717, Oujda 60000, Morocco; tikent.aziz@ump.ac.ma; 6 Oriental Center for Water and Environmental Sciences and Technologies (COSTE), Mohammed Premier University, Bp 717, Oujda 60000, Morocco; ayoub.farihi@uit.ac.ma; 7 Biological Engineering Laboratory, Faculty of Sciences and Techniques, Sultan Moulay Slimane University, Beni Mellal 23000, Morocco; laaroussi.meriem1@gmail.com (M.L.); mohamed.bouhrim@gmail.com (M.B.); f.chigr@usms.ma (F.C.); 8 Department of Pharmacognosy, College of Pharmacy, King Saud University, Riyadh 11451, Saudi Arabia; ashahat@ksu.edu.sa (A.A.S.); rherqash@ksu.edu.sa (R.N.H.); 9 Fruit and Vegetable Storage and Processing Department, The National Institute of Horticultural Research, Konstytucji 3 Maja 1/3, 96-100 Skierniewice, Poland; younes.noutfia@inhort.pl

**Keywords:** immature carob pulp, phytochemical compounds, antioxidant activity, in silico studies, in vitro studies, in vivo studies, antidiabetic agent

## Abstract

*Ceratonia siliqua* L. is a medicinal plant that has long been used in traditional Moroccan medicine to treat many diseases. This study aimed to assess the impact of the stages of the immature phase of carob pulp (M1, M2, M3, M4, and M5) on phytochemical composition, antioxidant activity, and antidiabetic activity of *Ceratonia siliqua* L. The identification of the phenolic profile by HPLC-UV/MS-MS and the study of the antidiabetic effect by in silico, in vitro, and in vivo studies were carried out for extracts with high contents of phenolic compounds from immature wild carob pulp from the communes of Timoulit (TM), Bin Elouidane (AW), and Ouaouizerth (TG) in the province of Azilal in the Béni Mellal-Khénifra region. The results revealed a gradual increase in total sugar content over the pulp’s ripening period, reaching a value of 2134 ± 56.23 mg GE/100 g fresh weight (FW) for TG. The three locations showed peak values for total polyphenol content (TPC), total flavonoid content (TFC), and total condensed tannin (TCT) at the M2 stage. AW had the highest concentrations of TPC (3819 ± 226.4 mg GAE/100 g FM), TFC (1034 ± 57.08 mg QE/100 g FM), and TCT (1472 ± 28.46 mg CE/100 g FM). The DPPH assay (7892 ± 296.1 mg TE/100 g FM) and the FRAP assay (278.2 ± 7.85 mg TE/100 g FM) both demonstrated that the TG zone is a highly potent antioxidant zone. In contrast, the AW site exhibited a markedly elevated value of 725.4 ± 103.6 mg TE/100 g FM in the ABTS assay. HPLC-UV-MS/MS analysis showed that the methanolic extracts of immature carob pulp (MEICP) from the three areas contained several different chemical compounds. The most prevalent were 3-O-p-coumaroyl-5-O-caffeoylquinic acid, quercetin 3-methyl ether, gallic acid, and galloylquinic acid. Immature carob pulp extract (ICPE) from AW showed the strongest in vitro inhibition of pancreatic α-amylase (IC_50_ = 0.405 µg/mL) and TG extracts were most potent against intestinal α-glucosidase (IC_50_ = 0.063 µg/mL). In vivo, AW, TG, and TM extracts significantly reduced postprandial glycemia in rats, with AW having the greatest effect. These results highlight the antidiabetic potential of ICPE. The 3-O-p-Coumaroyl-5-O-caffeoylquinic acid showed better affinity for α-amylase compared to acarbose and interacted significantly with several amino acid residues of the enzyme. Similarly, this molecule and 3,4-Dicaffeoylquinic acid demonstrated a strong affinity for α-glucosidase, suggesting their potential as natural inhibitors of enzymes involved in carbohydrate metabolism. Most of the compounds are not substrates of P-glycoprotein and exhibited high intestinal absorption. Furthermore, the majority of these compounds did not act as inhibitors or substrates of CYP450 enzymes, reinforcing their suitability for development as oral medications. These results underscore the potential of immature carob pulp as a promising antidiabetic agent.

## 1. Introduction

*Ceratonia siliqua* L. is a native tree found in the Mediterranean and Middle Eastern regions. It belongs to the Leguminosae family and is commonly called the carob tree. This plant is classified as an evergreen and can thrive in its natural habitat or when intentionally cultivated. It has a high level of tolerance towards various soil types. This species is essential for preventing soil erosion, degradation, and desertification due to its deep roots, evergreen leaves, and unique agroecological properties [1,2].

The carob tree’s fruit has recently attracted significant attention, evident from the publication of numerous research publications in the fields of food science and technology that specifically examine food products generated from the carob fruit. These possess exceptional characteristics, including their low-fat composition, abundant dietary fiber, minerals, and a significant quantity of polyphenols [3,4,5].

On average, the pulp and seeds make up 90% and 10% of the weight of a carob tree fruit, respectively. The chemical composition of the pulp exhibits significant variations based on factors such as the cultivar or species, climate, place of origin, and the stage of ripeness during harvest [6,7]. The pulp and seeds are frequently differentiated based on their high concentration of polyphenolic compounds, which have advantageous impacts on human well-being [8]. The high sugar content of the pulp makes it a valuable product in the culinary industry, where it is utilized to produce syrups and as a chocolate alternative in cookies and ice cream formulations [9,10,11]. The presence of dietary fiber in it confers numerous advantageous benefits on blood sugar, cholesterol levels, and cancer, making it suitable for use in the pharmaceutical industry [12].

The seed contains a substantial proportion of galactomannan (80–85%) [13], a widely used food additive (E410), that plays a vital role in food technology as it can be utilized as a stabilizer, thickener, and gelling agent. In addition, it finds application in the manufacturing of cosmetics, paint, textiles, and pharmaceuticals [14,15,16]. The carob fruit is a rich source of natural antioxidants that effectively protect against reactive oxygen species, including both free radicals and non-free radicals. Oxidative stress, resulting from oxidation processes affecting proteins, lipids, and nucleic acids, has been linked to several health problems, including cardiovascular disease, renal disease, and degenerative disease [17].

Polyphenols present in the carob pulp have been demonstrated to exert a range of physiological effects, including improvements in digestive function [18] and reductions in both blood cholesterol [19] and glucose levels [20]. An increase in glucose levels leads to oxidative stress, which in turn increases the non-enzymatic glycation of proteins and glucose oxidation [21]. The efficacy of medications such as sulfonylurea and biguanides in managing diabetes and mitigating its long-term complications has been inadequate [22].

Various medicinal plants and their extracts have shown efficacy in treating diabetes, including the carob fruit. The carob fruit stands out due to its abundant concentration of antidiabetic compounds and antioxidants, specifically flavonoids and gallotannins [23]. According to previous investigations, unripe carob fruits have higher levels of phenolic acids, flavonoids, and tannins compared to ripe fruits. They also exhibit higher levels of in vitro antioxidant activity [24,25,26,27,28]. Regarding carbohydrate content, unripe fruit has lower levels of sugars, whereas ripe fruit has higher amounts of sucrose [29].

Rtibi et al. (2017) found that immature carob pulp aqueous extract exhibits antidiabetic properties. Their findings indicate that the extract inhibits intestinal glucose transport, thereby reducing blood glucose levels [30].

Based on this information, we proceeded to study the effect of immature stages on the phytochemical composition of unripe carob pulp to consider their use in the pharmaceutical field. The objective of this study was to ascertain the levels of total sugars, total polyphenol content, total flavonoid content, and total condensed tannins in immature carob pulp from the Béni Mellal-Khénfra region in Morocco and to evaluate its antioxidant activity. Secondly, the elevated polyphenol content prompted the identification of the respective phenolic profiles and an investigation into the antidiabetic effects of immature carob pulp during this stage. This was achieved by utilizing in silico, in vitro, and in vivo studies.

## 2. Materials and Methods

### 2.1. Chemical Reagents

All chemicals used were analytical reagent-grade and did not require additional purification. Sulfuric acid (95–97%), gallic acid (97.5–102.5%), vanillin, hydrochloric acid (37%), and 2,4,6-Tris(2-pyridyl)-s-triazine (TPTZ); α-amylase enzyme quality level (300), starch quality level (200), acarbose pharmaceutical primary standard, α-glucosidase enzyme quality level (200), sucrose quality level (100), and GOD-POD (glucose oxidase–peroxidase) quality level (100); formic acid; and pure reference standards (P-coumaric acid, naringin (≥95%), rutin hydrate (≥94%, HPLC), 4-hydroxybenzhydrazide (≥97%), gallic acid, caffeic acid, chlorogenic acid hemihydrate, ferulic acid, and catechin) were purchased from Sigma-Aldrech (Darmstadt, Germany). Folin–Ciocalteu phenol reagent 2.0 and ferric chloride 99% (FeCl_3_) were purchased from the Oxford Range of Laboratory Chemicals (Maharashtra, India). Acetic acid and potassium persulfate, were purchased from VWR International S.A.S. (Fontenay-Sous-Bois, France). Methanol ≥ 99.8% (for liquid chromatography), ethanol absolute 99.9%, and phosphate buffer (Na_2_HPO_4_) ≤ 0.01%, were purchased from Merck (Darmstadt, Germany). Aluminum chloride (AlCl_3_) and phenol 99%, were purchased from Loba Chemie (Mumbai, India). Sodium carbonate, sodium acetate, and quercetin dihydrate 98% were purchased from HiMedia Laboratories Pvt Ltd. (Mumbai, India). 2,2-Diphenyl-1-picrylhydrazyl (DPPH) 95%, 2,2-diphenyl-1-picrylhydrazyl (DPPH), 2,2′-azino-bis-3-ethylbenzothiazoline-6-sulphonic acid (ABTS), 6-hydroxy-2, 5, 7, 8-tetramethylchroman-2-carboxylic acid (Trolox) 97%, and 3,5-dinitrosalicylic acid (DNSA) 98% were acquired from Thermo Fisher Scientific (New Jersey, USA).

### 2.2. Plant Materials Harvest

Immature carob pods were collected from three different areas in the Béni Mellal-Khénifra region of Morocco. Timoulilte commune (collection zone: Tanzight, abbreviated as TN; coordinates: 32°11′52.9″ N 6°19′52.2″ W), Bin Elouidane commune (collection zone: Ait-Waada, abbreviated as AW; coordinates: 32°08′41.1″ N 6°30′04.8″ W), and Ouaouizerth commune (collection zone: Tizi Ghnim, abbreviated as TG; coordinates: 32°12′24.9″ N 6°23′30.8″ W). Sampling was carried out between April and June 2022 to track the immature stage of the carob pods. Identification of the plant was performed by a professional botanist, Professor Fennane Mohammed from the Scientific Institute in Rabat, Morocco. Voucher specimens of *Ceratonia siliqua* L. were deposited in the Herbarium of Mohammed Premier University, Oujda, Morocco (HUMPOM74). Based on pod dimensions, five different stages (M1, M2, M3, M4, and M5) determined the time of harvest at the immature stage. Thus, ten trees from each area were selected and a total of 50 carob pods were systematically obtained from each tree by collecting an equivalent number of samples from all four cardinal directions (north, south, east, and west) and within the tree itself, with a careful selection process to ensure that only ripe and disease-free carob pods were sampled.

### 2.3. Preparation of the Immature Carob Juice

On arrival at the laboratory, the collected fruits were separated from the seeds to extract only the pulp, which was then crushed using an electric juice extractor (Taurus, model: Liquajuice LS-651BM, typ: SBL-1702D, Barcelona, Spain). The collected juice was centrifuged at 4500 rpm (2150× *g*) for 15 min (Sigma 2-16P, Sigma Laborzentrifugen GmbH, Osterode am Harz, Germany). The supernatant obtained was stored in opaque containers in the refrigerator to avoid oxidation. The carob juice was used for compositional analysis.

### 2.4. Quantification of Total Sugar Content

The Dubois et al. (1956) method was used to quantify the amounts of sugars in the juice [31]. A mixture was prepared by adding 1 mL of diluted juice (1/100) (*v*/*v*), 5 mL of sulfuric acid, and 1 mL of 5% phenol. After shaking, the mixture was left to stay in a dark environment for almost 45 min. The optical density was then measured at 488 nm using a Shimadzu UV-2401 PC UV spectrophotometer (serial N° A 10834232128CS, Suzhou instruments manufacturing, Suzhou Jiangsu, made in China). The results were expressed as mg of glucose equivalent per 100 g of carob fresh matter (mg GE/100 g FM). Three replicates were conducted for the same treatment.

### 2.5. Quantification of Total Phenolic Content

A volume of 500 μL of the diluted juice was added to a test tube. Subsequently, 2.5 mL of Folin–Ciocalteu reagent (10%) and 2 mL of sodium carbonate, 7.5% solution, were added at a two-minute interval. The samples were then maintained in a dark environment at a standard laboratory temperature for approximately 90 min before measuring their absorbance at a wavelength of 760 nm. The amount of total phenolic contents was calculated by using a standard curve and was expressed as mg gallic acid per 100 g fresh matter (FM) [32]. The measurements were performed in triplicate.

### 2.6. Quantification of Flavonoid Content

The flavonoid content was assessed using the aluminum chloride (AlCl_3_) procedure [33]. A mixture of 1 mL of carob juice and 1 mL of 2% AlCl3 was prepared and stored in a dark environment at lab temperature for almost 10 min. Then, the absorbance was carefully measured at 430 nm. Following a comparison with the standard curve, the flavonoid content was calculated and expressed as mg quercetin per 100 g FM. Measurements were carried out in triplicate for accuracy.

### 2.7. Quantification of Total Condensed Tannins

To determine condensed tannins present in the carob juice, we adapted the method developed by Broadhurst and Jones (1979) [34]. A solution of 400 µL of diluted juice (1/10) (*v*/*v*) was prepared, with a maximum of 3 mL of 4% vanillin methanol solution added. Subsequently, 1.5 mL of 37% concentrated hydrochloric acid was added. The mixture was then left to stand in the dark at room temperature for approximately 15 min, after which absorbance was measured at 500 nm against a blank consisting of methanol. The results are expressed as mg catechin equivalent (CE)/100 g FM using the catechin calibration curve. To ensure consistency, each measurement was performed three times.

### 2.8. Antioxidant Activity Evaluation Assays

#### 2.8.1. 2,2-Diphényl 1-Picrylhydrazyle Assay

The antioxidant capacity of immature carob pulp juice was determined using the 2,2-diphenyl-1-picrylhydrazyl (DPPH) assay, by the methodology previously established by Szabo et al. (2019) [35]. Specifically, 0.1 mL of juice was combined with ethanol/water (6:4) at a ratio of 1/1000 (*v*/*v*) and 2 mL of a 0.1 mM DPPH solution. The mixture was shaken and then incubated in complete darkness for approximately 45 min. The absorbance of DPPH was then measured at a specified wavelength of 517 nm. For comparison, a standard of Trolox (1–5 mg/mL) was employed (R² = 0.9706), and the final values of free radical scavenging activity of juice samples were expressed as milligrams of Trolox equivalents per 100 g FM. Measurements were performed in triplicate.

#### 2.8.2. ABTS Scavenging Assay

Antioxidant capacity was determined by performing the 2,2′-azino-bis (3-éthylbenzothiazoline-6-sulfonate) (ABTS) assay by the protocol established by Uysal et al. (2016) [36]. Initially, a 7 mM ABTS solution was combined with 2.45 mM potassium persulfate and incubated in the dark at room temperature for 12–16 h. Before the commencement of the experiment, the solution was diluted with ethanol to achieve an absorbance of 0.700 ± 0.02 at 734 nm. To ascertain the ABTS scavenging activity in milligrams of Trolox equivalents per 100 g of FM, 1 mL of extract was combined with 2 mL of ABTS solution and incubated at room temperature for 30 min. The absorbance was then measured at 734 nm. To ensure accuracy, triplicate measurements were conducted.

#### 2.8.3. Ferric Reducing/Antioxidant Power Assay (FRAP)

In their study, Benzie and Strain (1996) describe the preparation of the FRAP reagent, which we created by combining 0.3 M acetate buffer (pH 3.6), 10 mmol/L TPTZ, and 40 mmol/L FeCl3 in a 10:1:1 ratio [37]. The juice was combined with 3 mL of the freshly prepared FRAP reagent, incubated for 30 min, and vortexed. The absorbance was measured at 593 nm, and the results were expressed in milligrams of Trolox equivalents per 100 g of FM, using Trolox as a standard from 1 to 5 mg/mL (R² = 0.9706). Triplicate measurements were performed to ensure accuracy.

### 2.9. Identification of Unripe Carob Pulp Juice Phytochemical Compounds

#### 2.9.1. Solubilization of Polyphenols of Unripe Carob Pulp Juice

Polyphenols were extracted using methanol [38]. The juices extracted from unripe carob pulp were subjected to vacuum dehydration and then transformed into powder using a vacuum oven (Memmert UM 400, Nuremberg, Germany). Five grams of immature carob pulp powder in fifty milliliters of aqueous methanol (70%, *v*/*v*) was prepared and left for 24 h to obtain the methanolic extracts of immature carob pulp (MEICP).

#### 2.9.2. HPLC-UV-MS/MS Analysis

Extracts with high phenolic compound content from TM, AW, and TG unripe carob pulps were analyzed qualitatively using a liquid chromatography system coupled with a triple quadrupole mass spectrometer (Thermo Fisher Scientific, San Jose, CA, USA). A Kinetex C18 reversed-phase column (100 × 4.6 mm, 2.6 μm particles) was employed, with a gradient separation of solvent A (0.1% formic acid aqueous solution) and solvent B (methanol), as described in the study of Bouaouda et al. (2023) [39]: 0–3 min, linear gradient from 5% to 25% B; 3–6 min, at 25% B; 6–9 min, from 25% to 37%B; 9–13 min, at 37% B; 13–18 min, from 37% to 54% B; 18–22 min, at 54% B; 22–26 min, from 54% to 95% B; 26–29 min, at 95% B; and 29–29.15 min, returning to initial conditions at 5% B, and re-stabilizing the column from 29.15 to 31 min to the initial gradient 5% B. A concentration of 1 mg/mL of each standard was analyzed with the same conditions. The identification of phenolic compounds relied on comparing retention time and spectra with nine standards, along with referencing the NIST-MS/MS library.

### 2.10. Animals

This study was conducted on albino mice (male and female). The animals were raised in the animal facility of the Department of Biology at the Faculty of Science and Technology of Béni Mellal, with a photoperiod of 12 h of light/12 h of darkness and a temperature of 22 ± 2 °C. The animals were kept in favorable breeding conditions with free access to water and food. All rats were cared for in compliance with the internationally accepted *Guide for the Care and Use of Laboratory Animals*, published by the US National Institutes of Health, 2011 [40].

### 2.11. Acute Toxicity Test

For each sample, two groups of fasting albino mice (25–35 g, 14 h) were divided into four groups (*n* = 6; ♂/♀=1): a control group gavaged with distilled water (10 mL/kg) and three treated groups gavaged with plant extract at doses of 2 g/kg, 1 g/kg, or 0.5 g/kg. At the beginning of the experiment, the mice were weighed and then gavaged with a single dose of the plant extract. They were monitored continuously for 10 h to detect any obvious signs of toxicity, and then daily for 14 days to observe any additional clinical or behavioral signs of toxicity.

### 2.12. Evaluation of the Inhibitory Effect of Unripe Carob Pulp on Pancreatic α-Amylase Activity

#### 2.12.1. In Vitro Assay

To investigate the in vitro inhibition of pancreatic α-amylase, we conducted antidiabetic tests on digestive enzymes using extracts from the stages that yielded the greatest concentrations of polyphenols and flavonoids, along with the most substantial antioxidant effects. This approach was based on an adapted version of the procedure outlined by Elrherabi et al. (2023) [41]. The test mixtures were prepared by combining 200 µL of a 0.02 M phosphate buffer solution (pH 6.9) and 200 µL of α-amylase enzyme solution, and incubating the mixture at 37 °C for 10 min. Subsequently, 200 µL of TG, TM, and AW solutions (at concentrations of 0.062, 0.125, 0.25, 0.5, and 1 mg/mL) were added to the respective tubes. Following this, 200 µL of a 1% starch solution was added to each tube, and the mixtures were incubated at 37 °C for 15 min. To terminate the enzymatic reaction, 600 µL of 3,5-dinitrosalicylic acid (DNSA) reagent was added, and the tubes were heated in an incubator at 100 °C for 8 min. After this thermal treatment, the tubes were cooled in an ice-water bath, and 1 mL of distilled water was added to dilute the samples. The absorbance of the samples was recorded at 540 nm with a spectrophotometer. To measure 100% enzyme activity, a blank was prepared by replacing the extract with 200 µL of phosphate buffer. Additionally, another blank was set up by applying the plant extract at various doses without adding the enzyme solution. Acarbose was employed as a positive control at comparable concentrations, with the reaction process mirroring that of the plant extract. The inhibition ratio was then computed using the formula provided below:

$$\%\;\mathrm{of}\;\mathrm{Inhibition}=\frac{A\;\mathrm{control}\;-\;A\;\mathrm{sample}}{A\;\mathrm{control}}\times 100$$

A_Control_: Absorbance of enzymatic effect in the absence of an inhibitor.

A_Sample_: Absorbance of enzymatic effect in the presence of extracts or acarbose.

The IC_50_ (concentration of samples that inhibit 50% of α-amylase enzyme activity) was calculated graphically using the following formula: percentage of inhibition = f (log (sample concentration)).

#### 2.12.2. In Vivo Assay

The oral starch tolerance test was utilized to evaluate the antihyperglycemic effect of the unripe carob pulp in vivo. Normal mice were divided into 3 groups (n = 6; ♂/♀ = 1). Control group: normal mice administered with distilled water (10 mL/kg); test group: normal mice administered with the plant aqueous extract (400 mg/kg); medication group: normal mice administered with acarbose (10 mg/mL). The test was conducted as follows: A blood glucose measurement was taken at t_0_. Immediately afterward, the test product (ED, the aqueous extract, or acarbose) was administered. Thirty minutes later, a second blood glucose measurement was taken, and then the mice were orally loaded with starch (3 g/kg). Subsequently, the variation in blood glucose levels was monitored for 2 h at 60, 90, and 120 min.

### 2.13. Assessment of the Inhibitory Effect of Unripe Carob Pulp on Intestinal α-Glucosidase Activity

#### 2.13.1. In Vitro Assay

The methodology for studying the inhibition of α-glucosidase activity adhered to the method described by Elrherabi et al. (2023b) [41]. The assay mixtures consisted of 1 mL of phosphate buffer (pH = 7.5), 0.1 mL of α-glucosidase enzyme solution (10 IU), and 200 µL of TG, TM, or AW (at concentrations of 0.031, 0.062, 0.125, 0.25, 0.5, and 1 mg/mL), with a corresponding volume of distilled water. Acarbose (at concentrations of 0.041, 0.082, 0.165, 0.328, and 0.656 mg/mL) served as the control, including appropriate negative and positive controls. The mixtures underwent pre-incubation for 20 min at 37 °C. Subsequently, 0.1 mL of sucrose was introduced to each tube, and the reaction was halted by incubating the tubes at 100 °C for 5 min. Following this, 1 mL of GOD-POD (glucose oxidase–peroxidase) was added, and the tubes were further incubated at 37 °C for 10 min. The absorbance was recorded at 500 nm using a spectrophotometer. The inhibition ratio was calculated using the following formula:

$$\%\;\mathrm{of}\;\mathrm{Inhibition}=\frac{A\;\mathrm{control}\;-\;A\;\mathrm{sample}}{A\;\mathrm{control}}\times 100$$

A_control_: Absorbance of enzymatic activity without the inhibitor.

A_sample_: Absorbance of enzymatic activity in the presence of the extract or acarbose.

The concentration of samples required to inhibit 50% (IC_50_) of α-amylase enzyme activity was determined graphically using the following formula: inhibition percentage = f (log sample concentration).

#### 2.13.2. In Vivo Assay

The oral sucrose tolerance test was employed to evaluate the antihyperglycemic impact of the immature carob pulp in vivo. Normal mice were segregated into 3 groups (n = 6; ♂/♀ = 1). Control group: normal mice administered with distilled water (10 mL/kg); test group: normal mice administered with the plant aqueous extract (400 mg/kg); medication group: normal mice administered with acarbose (10 mg/mL). The test was conducted as follows: a blood glucose measurement was taken at t_0_. Immediately afterward, the test product (ED, the plant aqueous extract, or acarbose) was given. After thirty minutes, a second blood glucose measurement was taken, and then the mice were orally loaded with sucrose (2 g/kg). Subsequently, the variation in blood glucose levels was monitored for 2 h at 60, 90, and 120 min.

### 2.14. Molecular Docking Analysis

#### 2.14.1. Ligand Preparation

The phytochemicals detected by HPLC-UV-MS/MS in methanolic extracts of immature carob pulp (ICPME), as well as the drug acarbose (CID: 41774) (a standard inhibitor), were sourced in 3D SDF format from the PubChem database (available at https://pubchem.ncbi.nlm.nih.gov/ (accessed on 12 June 2024)). However, the 3D model of 3-O-p-coumaroyl-5-O-caffeoylquinic acid was created using Avogadro 1.2 software. To assess the potential of these ligands as inhibitors of intestinal α-glucosidase and pancreatic α-amylase, the ligands were first converted to PDB format using PyMOL Molecular Graphics System (version 2.5.3) [42], and then further processed to PDB format using AutoDockTools (ADT; version 1.5.7, The Scripps Research Institute) [43]. Visualization of the molecular interactions between the ligands and receptors, including their binding affinities compared to the standard inhibitor, was achieved with Discovery Studio Visualizer (Biovia, 2021), aiding in the generation of the graphics [42].

#### 2.14.2. Protein Preparation

The crystal structures of human α-glucosidase (PDB ID: 5NN8) and α-amylase (PDB ID: 1B2Y) were obtained from the Protein Data Bank (PDB) (accessible at www.rcsb.org (accessed on 12 June 2024)) [44]. Each crystal structure was individually processed by removing water molecules and adding polar hydrogens and Kollman charges using AutoDockTools (ADT; version 1.5.7). For molecular docking, a grid with a point spacing of 0.375 Å and dimensions of 40 × 40 × 40 was created [45]. This grid was positioned to cover both the peripheral regions and active sites of the proteins, based on the x, y, and z coordinates. The processed macromolecules were then saved in PDB format for further molecular analysis.

### 2.15. ADME Studies

Understanding pharmacokinetic properties, such as absorption, distribution, metabolism, and excretion (ADME), is vital for determining how a substance behaves in the body [46]. These stages describe the path a substance takes from its absorption to its elimination. Computational tools have become indispensable for predicting the ADME characteristics of molecules. They allow for the evaluation of a molecule’s ability to cross cellular barriers, interact with key transporters and enzymes involved in absorption and excretion, and assess its metabolic stability [47]. In our molecule evaluation process, we opted to use the SwissADME platform. This platform allows for a comprehensive analysis of the physicochemical properties of phytochemicals identified in methanolic extract of unripe carob pods, their potential as therapeutic agents, and their pharmacokinetic characteristics, providing a thorough understanding of their ADME profile [47].

### 2.16. Statistical Analysis

All data are expressed as mean ± standard deviation (mean ± SD) of three determinations. Statistical analysis was performed using the one-way ANOVA method in GraphPad PRISM 8.0.1 software. Using Duncan’s multiple-range test, the mean values were separated with a level of significance as *p* < 0.05.

## 3. Results

### 3.1. Total Sugar Content

Table 1 presents the total sugar content at different stages of maturity harvested in different areas of the province of Azilal, Morocco. During the immature stage, the sugar content of all sources increased, with the highest amounts observed in TG and AW at M5 (2134 ± 56 mg GE/100 g FM and 2098 ± 34 mg GE/100 g FM, respectively). Nevertheless, M3 exhibited the highest TM value (1987 ± 72 mg GE/100 g FM). The overall sugar analysis revealed no statistically significant differences (*p* < 0.05) between TG, TM, and AW, except for the M3 stage, where TG differed from TM and AW. However, during the immature stage, we observed significant differences among the zones themselves, the variation in total sugar content across the three regions may be attributed to variations in their geographical provenance, meteorological factors, and soil properties [48].

To the best of our knowledge, no research has been conducted to specifically examine the overall sugar content of fresh unripe carob pulp.

Vekiari et al. (2012) conducted research that demonstrated a substantial increase in the sugar content of two different types of maturing Greek carob pods [49]. As stated by Prasanna et al. (2007), the rise in sugar content and sweetness is a natural occurrence during the maturation of numerous fruits. The fruit softens due to the enzymatic breakdown of cell wall polysaccharides through hydrolysis [50]. The low sugar content of fruit can be beneficial for blood sugar management, which is essential for people with diabetes. Furthermore, a study conducted on the polysaccharides in baobab fruit demonstrated that they possess notable antidiabetic properties by inhibiting enzymes such as α-amylase and α-glucosidase, which are involved in the breakdown of carbohydrates. This inhibition results in a slower release of glucose into the bloodstream, facilitating the regulation of blood sugar levels [51].

### 3.2. Total Phenolic, Flavonoid, and Condensed Tannin Contents

Table 2 presents the results for bioactive compounds in immature carob pulp, with a focus on total polyphenol content (TPC), total flavonoid content (TFC), and total condensed tannins (TCT) for the three zones (TG, TN, and AW) in the five immature stages. The TPC for TG, TN, and AW ranged from 2532 ± 48 to 3475 ± 189 mg GAE/100 g FM, 2120 ± 151 to 3819 ± 226 mg GAE/100 g FM, and 1947 ± 55 to 2765 ± 144 mg GAE/100 g FM, respectively. The M2 stage demonstrated the greatest TPC value among the three zones tested, whereas the M1, M5, M3, and M4 stages exhibited TPC values in a declining order.

The statistical analysis revealed a significant decrease (*p* < 0.05) in the total phenolic content of TG, TN, and AW from M1 to M3. However, there was a slight increase in AW from M4 to M5. It is important to mention that there was considerable variation between zones for each stage, except for the M1 stage.

Gull et al. (2012) found that the decrease in polyphenolic content in carob fruit is due to the oxidation of phenolic compounds by polyphenol oxidase as the fruit approaches maturity. This leads to the conversion of soluble phenols into insoluble phenolic compounds that bind to the cell walls’ polysaccharides [52]. Wang et al. (2018) suggest that the reduction in phenolic content may be attributed to the instability of their structures, which were transformed into other compounds throughout the biosynthesis process. Additionally, the authors postulated that the alteration of enzymatic activity during the biosynthesis process in *Ribes stenocarpum* Maxim fruit constituted a significant factor. Nevertheless, the precise mechanism remains elusive, underscoring the necessity for further investigation [53].

The TFC results demonstrated that the TG values ranged from 173.2 ± 11 to 925.4 ± 13 mg QE/100 g FM, while the TN values exhibited a broader range, from 196.2 ± 11 to 1034 ± 57 mg QE/100 g FM. AW values exhibited a similar range, spanning from 161.2 ± 13 to 1423 ± 78 mg QE/100 g FM.

The results indicate a notable decline in flavonoid levels from M1 to M4, followed by a slight increase at M5 in all three studied zones. Furthermore, in the initial three stages, there is a significant difference (*p* < 0.05) in flavonoid levels among the three zones.

The role of flavonoids in protecting the photosynthetic apparatus may account for their greater abundance during the early stages of the immature phase compared to the final stage. According to Harborne and Williams (2000), flavonoids play a protective role in photosynthetic tissues against damage. When chlorophylls break down, the photosynthetic process ceases, leading to a decrease in TFC in the carob pod during ripening [54]. Flavonoids are also renowned for their antioxidant properties, which may assist in reducing the oxidative stress associated with diabetes. This diminished oxidative stress may facilitate enhanced insulin sensitivity and glucose metabolism. Furthermore, flavonoids may facilitate an increase in insulin secretion from pancreatic cells, thereby providing additional support for regulating blood sugar levels [51].

The total condensed tannins (TCT) analysis reveals that the juices extracted from the immature carob pulp of TG, TN, and AW have the highest concentrations at the M2 stage, with values of 848.1 ± 19, 1472 ± 28, and 756.1 ± 92 mg CE/100 g FM, respectively. On the other hand, the M5 stage yields the lowest concentrations, measuring 75.7 ± 5.5, 71.4 ± 3.3, and 77.1 ± 6.1 mg CE/100 g FM, respectively. The results show that TN has the highest concentration of condensed tannins, which indicates its potential role in a range of biological activities.

The concentration of condensed tannins decreased significantly as the carob fruit matured from the M1 to M4 stages, and there was a significant decrease at the M5 stage. Substantial polymerization and condensation processes are responsible for the decrease in tannin content during carob ripening [55].

The obtained results suggest that a number of factors, including cultivar, geographic origin [56], ecological conditions during harvesting, genotype, growth conditions, genetic diversity, and fruit maturity stage, may link to the variations in TPC, TFC, and TCT in immature carob pulp [55,57,58].

The high levels of polyphenols, flavonoids, and gallotannins in carob have demonstrated that immature carob can inhibit intestinal glucose absorption in addition to exerting other beneficial effects, including a potent antioxidant action [59].

The polyphenol, flavonoid, condensed tannin, and antioxidant activity content of fresh, unripe carob pulp has yet to be fully elucidated. Nevertheless, by drawing parallels with other fruits, our results indicate that the pulp of *Ceratonia siliqua* L. is a notable source of polyphenols. Fresh carob pulp, for example, has a higher total polyphenol content (TPC) than other fruits, including unripe plums (214.25 mg TPC/100 g fresh weight) [60]. This suggests that it may have a potential richness in bioactive substances at the immature stage.

### 3.3. Antioxidant Activities

**Figure 1:**
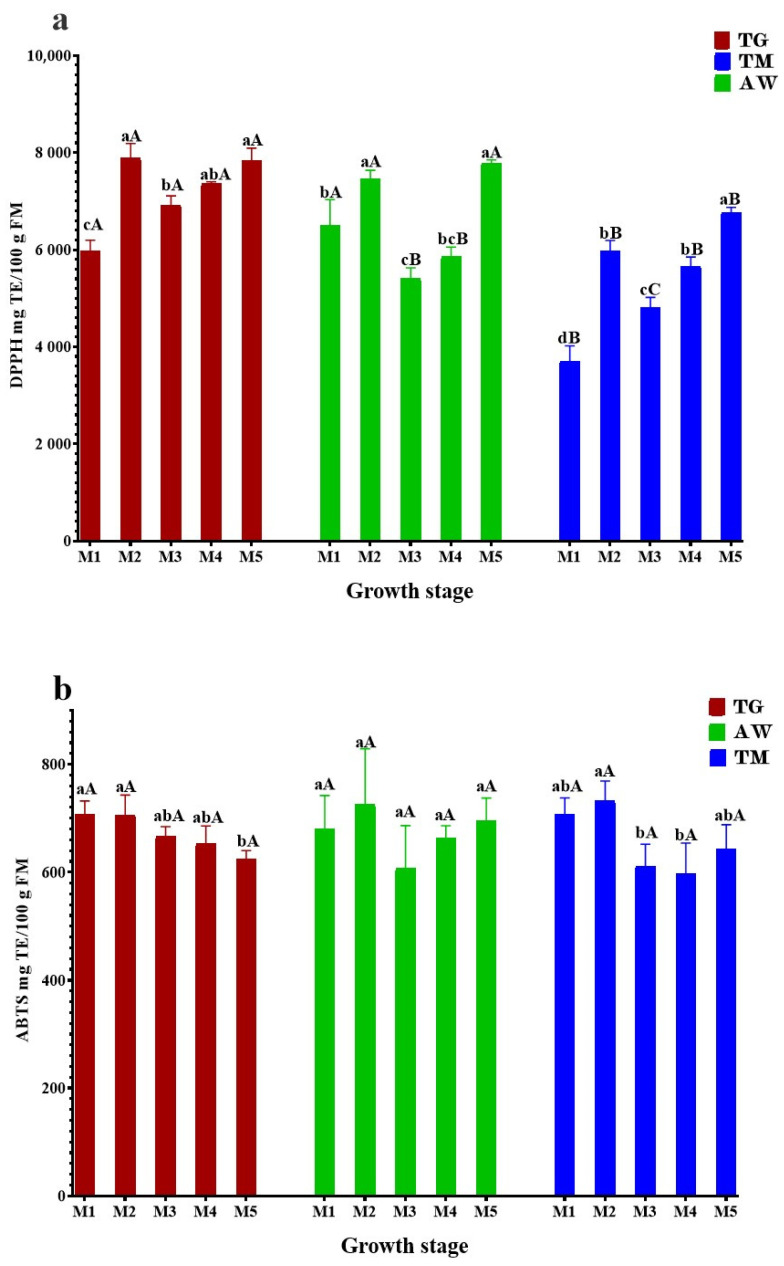

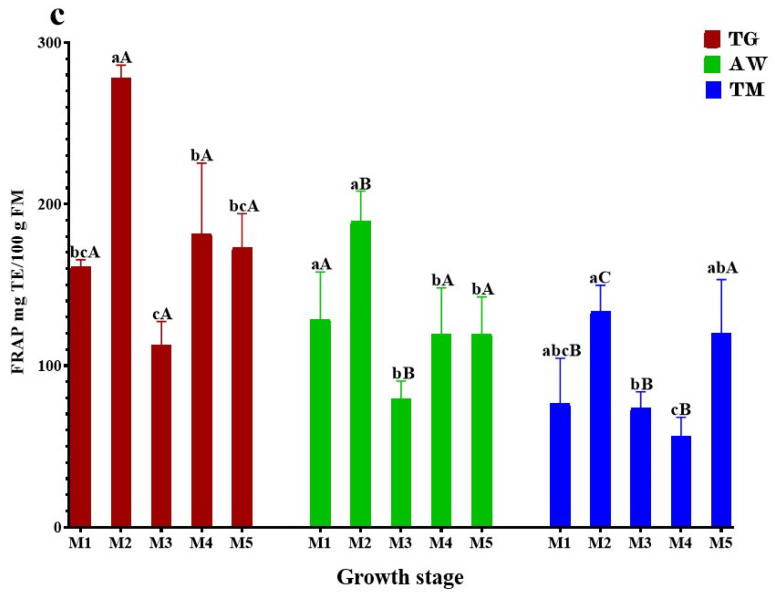
The antioxidant activity of unripe carob pulp during ripening stages using DPPH (**a**), ABTS (**b**), and FRAP (**c**) tests. The results are shown as the mean value ± SD (n = 3). Distinct lowercase letters indicate statistically significant variations (*p* < 0.05) across unripening phases, while distinct capital letters indicate statistically significant variations (*p* < 0.05) between provenances as determined by Tukey test. TG: Tizi-Ghnim site; TM: Timoulilt site; AW: Ait Waarda site; (M1, M2, M3, M4, and M5): stages of the immature phase of carob pulp; TE: Trolox equivalent; FM: fresh matter.

The main role of bioactive compounds in plant extracts is to prevent diseases and promote human health. These compounds have gained significant attention in recent years because of their ability to act as antioxidants. The results shown in Figure 1 demonstrate that the unripe carob pulp is proficient in efficiently trapping free radicals across all phases of analysis.

The highest DPPH antioxidant capacity of immature carob pulp for the three zones (TG, AW, and TM) (Figure 1a) was exhibited in stages M2 (7892 ± 296, 7458 ± 178, 5986 ± 205 mg TE/100 g FM) and M5 (7844 ± 249, 7786 ± 64, and 6772 ± 99 mg TE/100 g FM). The obtained values demonstrate a statistically significant difference (*p* < 0.05) primarily between the two zones’ TG, AW, and TN. The majority of the analyzed internships show a high degree of similarity between TG and TN.

According to Barku et al. (2013), plant antioxidant activity is dependent on the presence of phenolic compounds and the structure of these compounds [61]. The decrease in capacity to capture free radicals at stages M1, M3, and M4 can be attributed to the decline in phenolic compound levels caused by fruit growth. However, Mena et al. (2011) published that the difference in antioxidant activity is associated with the variation in phytochemical composition [62].

We also evaluated the plant extracts for their ability to scavenge free radicals using the ABTS radical decolorization assay. This assay is based on the reduction of ABTS+● radicals by the antioxidants present in the tested plant extracts. As depicted in Figure 1b, the outcomes were largely comparable to the DPPH activity, except TG, where the activity exhibited a decline after M3. Stage M2 had the highest amount of ABTS for TG, AW, and TM, with values of 705.7 ± 37, 725.4 ± 103, and 733.2 ± 35 mg TE /100 g FM, respectively. The lowest levels of ABTS were observed in M5 for TG (624 ± 16 mg TE/100 g FM), M3 for AW (607.4 ± 79 mg TE/100 g FM), and M4 for TM (598.1 ± 58 mg TE/100 g FM).

In acidic conditions, the FRAP assay checks how well antioxidants work at changing Fe^3+^ to Fe^2+^ [63]. We acquired the results using the Trolox calibration curve. The antioxidant capacity of TG ranged from 113 ± 14 to 278.2 ± 8 mg TE /100 g FM, AW varied between 79.5 ± 11 and 189.5 ± 18 mg TE /100 g FM, while the TM ranged from 56.3 ± 11 to 133.6 ± 16 mg TE /100 g of FM (Figure 1c). The TG, AW, and TM showed the highest reducing ability with values of 278.2 ± 8 mg TE /100 g FM, 189.5 ± 18 mg TE/100 g FM, and 133.6 ± 16 mg TE /100 g FM, respectively. Comparable to the results observed in DPPH assays.

According to Ferreira-Santos et al. (2024), the variations in antioxidant capacity observed in the three zones studied can be attributed to the chemical structure of the radical molecules and the specific phenolic compounds present in each residue [64]. In addition, variables such as residue diversity and ripening stage can also have an impact on antioxidant capacity [65]. In a study conducted by Richane et al. (2022), it was demonstrated that a number of phenolic compounds, including gallic acid, syringic acid, kaempferol, and apigenin, exhibited a notable correlation with antioxidant properties when evaluated in vitro. This suggests that these compounds are the primary contributors to the antioxidant activity of the pulp extract from *Ceratonia siliqua* L. [56]. Previous studies have documented the influence of gallic acid, isoquercetin, and apigenin on free radical scavenging activity [66]. The chemical interactions among the numerous major and minor phenolic components can alter the antioxidant activity of plant extracts [67].

Our results indicate that immature carob pulp from the three study sites has high antioxidant activity. Consequently, it may be a valuable source for the prevention or mitigation of diseases related to oxidative stress. The pronounced antioxidant activity of carob may be attributed to its high concentration of polyphenolic compounds. These findings confirm that carob has the potential to serve as a rich source of natural antioxidants, which may play a key role in maintaining health and preventing disease [59].

### 3.4. HPLC-UV-MS/MS Analysis

The methanolic extracts of immature carob pulp (MEICP) from the M2 stage were selected for study because of their high polyphenolic content. The HPLC-UV-MS/MS method was used to determine the phenolic profile and identify the phenolic compounds present in the MEICP from the three zones.

**Figure 2:**
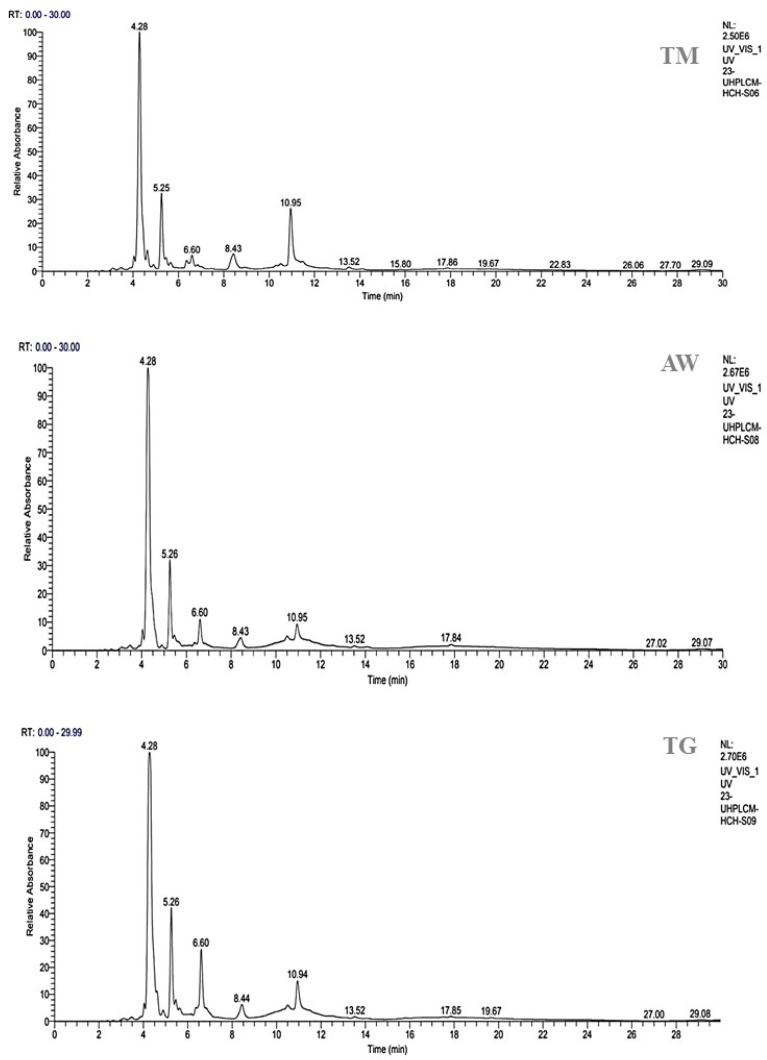
Phenolic profile of the TN, AW, and TG unripe carob pod sites. RT: retention time; TG: Tizi-Ghnim site; TM: Timoulilt site; AW: Ait Waarda site.

A total of seven peaks were identified through HPLC analysis as part of our investigation. 3-O-p-coumaroyl 5-O-caffeoylquinic acid, quercetin 3′-methyl ether, gallic acid, galloylquinic acid, and 3,4-dicaffeoylquinic acid were the main chemicals found in MEICP from all three areas (Table 3, Figure 2). When examining the individual components, 3-O-p-coumaroyl 5-O-caffeoylquinic acid has almost identical percentages in different locations. Specifically, TM and TG have percentages of 53.40% and 55.84%, respectively, whereas AW has a greater proportion, up to 63.62% of this acid. Quercetin 3-methyl ether is present at similar percentages in TM (13.87%), AW (12.91%), and TG (13.83%). Conversely, galloylquinic acid is only present in TM (12.55%). Gallic acid is particularly high in TM (11.24%), whereas it is relatively low in AW and TG (5.17% and 2.35%, respectively). All areas contain 3,4-dicaffeoylquinic acid, with TG exhibiting comparatively greater proportions than TM and AW. In their study, Can et al. (2020) investigated the protective effects of *Daphne gnidioides Jaub* and *Spach* extracts on DNA, their antibacterial properties, their potential in combating cancer, and their ability to inhibit enzyme activity. They concluded that significant amounts of vicenin-2 and 3-O-p-coumaroyl-5-o-caffeoylquinic acid in these extracts could be associated with these biological activities [68]. To our knowledge, there has been no research into the potential antidiabetic properties of this particular molecule. Several plants such as *Allagopappus viscosissimus* [69], *Opuntia ficus-indica var. saboten* [70], *Lychnophora staavioides* [71], and Rhamnus species contain the flavonoid querectin-3-methyl ether, which, to the best of our knowledge, has not previously been reported in carob pod. Studies have shown that quercetin-3-methyl ether has anti-inflammatory [72], antioxidant [73], significant anticancer-promoting [74], antitrypanocidal, antimutagenic, tracheal relaxing, radical scavenging, and xanthine oxidase-inhibitory properties [71]. The research conducted by Motta et al. (2017) discovered that galloylquinic acid, which was detected in zone TM (12.55%), was primarily responsible for the gastroprotective effect [75]. Furthermore, galloylquinic acid exhibited a considerable level of cytotoxicity against stomach cancer cells when tested in isolation [75]. TM extract contains substantial amounts of gallic acid, while TG and AW only contain minor amounts. Multiple studies have demonstrated that this compound, which is prevalent in various plant compounds, possesses antitumor properties [76] and acts as an antioxidant, safeguarding against a variety of diseases. This includes the prevention of neurodegeneration resulting from oxidative stress and other neuroprotective mechanisms. It is thought that gallic acid could be a useful first compound for treating several neurodegenerative diseases, such as brain tumors, glioblastoma, cerebral ischemia/reperfusion, anxiety, memory loss, depression, sedation, psychosis, neuropathic pain, Alzheimer’s disease, Parkinson’s disease, and neuroinflammation [77]. The variation in compound percentages indicates potential regional factors that may affect the chemical makeup of carob pods. In some areas, compounds such as galloylquinic acid and ferulic acid are noticeably absent or found in very small quantities, suggesting the specificity of their distribution. These findings demonstrate the significance of considering geographical variables when examining the chemical composition of natural extracts. They also give us useful information for future research on the bioactivity and possible uses of carob pods from various geographical origins [29].

### 3.5. Acute Safety

The results of the acute toxicity evaluation show that none of the three extracts demonstrated any toxicity, even at a dose of 2 g/kg. No toxic effects such as diarrhea, vomiting, or abnormal mobility were observed, and no deaths were recorded during the entire observation period (Table 4). A study by Moumou et al. (2023) investigated the acute toxicity of a single dose of immature locust bean pod extract at 2 and 5 g/kg body weight in mice. The results indicated that immature carob pod extract is non-toxic with an LD50 greater than 5 g/kg body weight [78].

### 3.6. In Vitro, Inhibitory Effect of the Plant Extract on Pancreatic α-Amylase and Intestinal α-Glucosidase Activities

#### 3.6.1. Pancreatic α-Amylase

Among the many digestive enzymes in humans, pancreatic α-amylase (EC 3.2.1.1) is particularly crucial. It drives the hydrolysis of α-1,4 glycosidic bonds in starch, facilitating its digestion. Since large starch molecules cannot cross the blood–brain barrier, α-amylase breaks them down into smaller sugar fragments that can enter the bloodstream and subsequently reach the brain. If starch is converted into sugars excessively, blood sugar levels rise, prompting insulin to instruct cells to metabolize and store the excess sugar as glycogen. This process maintains equilibrium in a healthy individual. However, excessive amylase activity coupled with insulin deficiency or resistance can lead to elevated blood glucose levels, potentially causing hyperglycemia. Inhibiting this enzyme can slow down the digestion of dietary polysaccharides; this process decreases the uptake of carbohydrates in the intestines and reduces post-meal blood sugar levels. Consequently, research into inhibiting amylase activity is ongoing to manage and control hyperglycemia [79].

**Figure 3:**
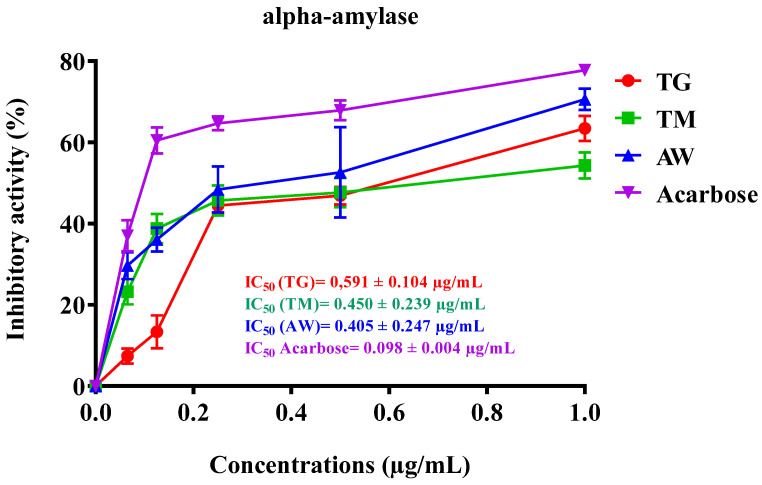
Inhibitory effect of TG, TM, AW, and acarbose on pancreatic α-amylase activity of human pancreatic cells in vitro. The results are shown as the mean value ± SD (n = 3). TG: Tizi-Ghnim site; TM: Timoulilt site; AW: Ait Waarda site; (M1, M2, M3, M4, and M5): stages of the immature phase of carob pulp.

The inhibitory effect on α-amylase activity observed in vitro by immature carob pulp extract (ICPE) from the three regions TM, AW, and TG, as seen in Table 5 and Figure 3, showed notable differences. The ICPE from AW demonstrated the most potent inhibition with an IC_50_ of 0.405 ± 0.247 µg/mL, followed closely by TM with an IC_50_ of 0.450 ± 0.239 µg/mL, and TG with an IC_50_ of 0.591 ± 0.104 µg/mL. Luísa Custódio et al. (2015) revealed that at a concentration of 1 mg/mL, decoctions from the leaves (74%) and stem bark (65%) of *C. siliqua* L. had potent α-amylase inhibition, surpassing the standard α-amylase inhibitor (acarbose) [80]. This is the first time we have assessed the impact of the stages of the immature phase of carob pulp (M1, M2, M3, M4, and M5) on digestive enzymes. We concentrated on the stages that produced the greatest quantities of polyphenols and flavonoids, along with the most significant antioxidant activity.

Germ flour exhibited moderate inhibition (40% at 1 mg/mL; 42% at 10 mg/mL), while locust bean gum and pulps had low or no activity [80]. A separate study reported that the aqueous extract of *C. siliqua* L. demonstrated a significant inhibitory effect on all digestive enzymes [81]. These findings are consistent with Custódio et al. (2015), who observed that both the leaves and stem bark of the carob tree exhibit strong inhibition of α-amylase and α-glucosidase activities, along with notable antioxidant properties [80]. Diabetes mellitus is a metabolic disorder often linked to increased cellular oxidative stress and diminished antioxidant activity. Natural antidiabetic compounds such as polyphenols and flavonoids mitigate free radical formation, decrease oxidative stress, and inhibit digestive enzymes, which in turn helps lower post-meal glucose levels [82]. The superior inhibitory activity observed in the AW and TM regions can be ascribed to their higher levels of total secondary metabolites, including catechin for AW, a known potent α-amylase inhibitor. Additionally, recent research often highlights the α-glucosidase-inhibitory effects of basic polyphenols, including phenolic acids and flavonoids.

We conducted this test to find traditional alternatives for treating diabetes because the initial phase of acarbose treatment may be linked with side effects such as bloating, gas, and diarrhea [83].

#### 3.6.2. Intestinal α-Glucosidase

**Figure 4:**
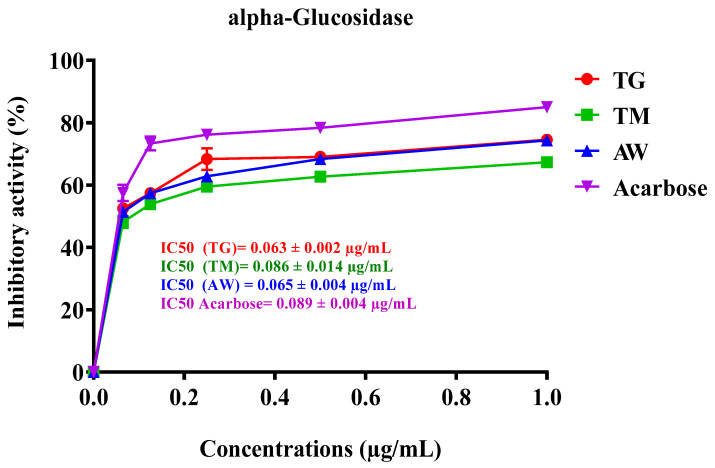
Inhibitory effect of TG, TM, AW, and acarbose on intestinal α-glucosidase activity of human pancreatic cells in vitro. The results are shown as the mean value ± SD (n = 3). TG: Tizi-Ghnim site; TM: Timoulilt site; AW: Ait Waarda site; (M1, M2, M3, M4, and M5): stages of the immature phase of carob pulp.

Intestinal α-glucosidase performs an essential function in the digestion of carbohydrates by breaking down starch and disaccharides into glucose, facilitating their absorption in the intestines. Inhibitors of α-glucosidase are considered key targets for developing new treatments for diabetes, as they can help manage blood glucose levels [84]. Polyphenol-rich plant foods have been documented to mimic insulin’s effects on glucose metabolism and serve as effective inhibitors of crucial enzymes like α-amylase and α-glucosidase, which are linked to type 2 diabetes and tissue lipid peroxidation [85]. In our study, we investigated the inhibitory effect of ICPE from three different regions (TG, TM, and AW) on α-glucosidase activity. The IC_50_ values demonstrated a strong inhibitory effect, with TG showing the most potent activity at 0.063 ± 0.002 µg/mL, followed by AW at 0.065 ± 0.004 µg/mL and TM at 0.086 ± 0.014 µg/mL (Table 6, Figure 4). These results are particularly significant relative to the standard inhibitor, acarbose, which has an IC_50_ of 0.089 ± 0.004 µg/mL. The extracts from TG and AW regions exhibit superior inhibitory effects on α-glucosidase, indicating a higher potential for managing postprandial blood glucose levels. This enhanced inhibition is likely due to the rich polyphenolic content, including compounds such as 3-O-p-coumaroyl 5-O-caffeoylquinic acid, quercetin 3-methyl ether, galloylquinic acid, and gallic acid, known for their enzyme inhibitory and antioxidant properties, as demonstrated in this study. A previous study demonstrated that the leaf and stem bark samples of the plant exhibited a potent inhibitory activity on α-glucosidase, with both samples showing 99% inhibition at a concentration of 10 mg/mL. This inhibition was significantly higher than that of acarbose, which showed an 85% inhibition [80]. It has been documented that hydrolyzable tannins have been shown to inhibit α-glucosidase activity. Moreover, *C. siliqua* germ is known to be rich in glycosylated derivatives of (iso)schaftoside. Apigenin 6,8-C-di-glycosides have also been identified as effective α-glucosidase inhibitors [86].

### 3.7. In Vivo, the Plant Extract’s Effect on Inhibiting Pancreatic α-Amylase and Intestinal α-Glucosidase Activities

#### 3.7.1. Pancreatic α-Amylase

**Figure 5:**
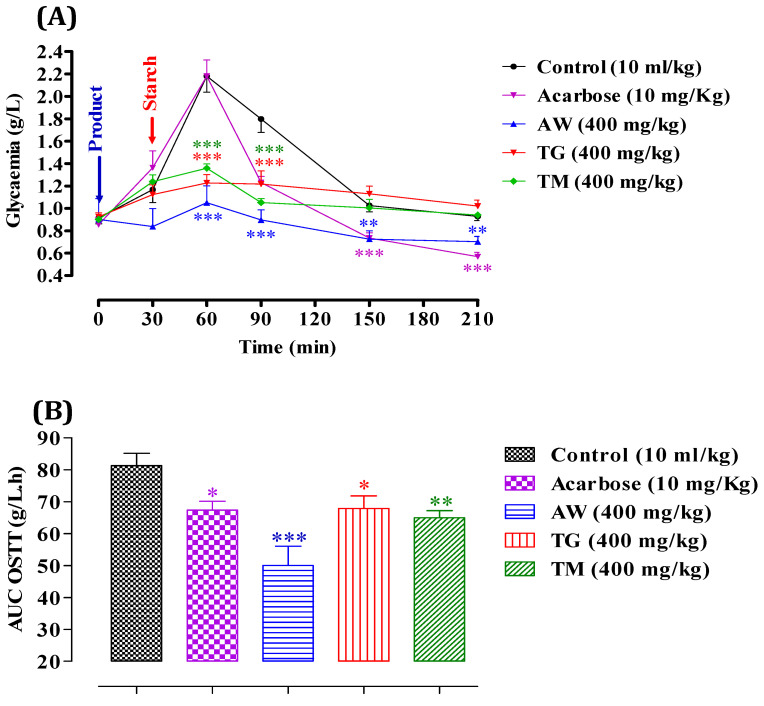
Impact of AW, TG, TM, and acarbose on glycemia following starch overload (2 g/kg) in normal rats (n = 6) (**A**). Area under the curve (AUC) for rats subjected to starch overload in the presence of AW, TG, TM, or acarbose (**B**). * *p* < 0.05; ** *p* < 0.01; *** *p* < 0.001. TG: Tizi-Ghnim site; TM: Timoulilt site; AW: Ait Waarda site.

We evaluated the inhibitory effect of ICPE on α-amylase in vitro, which demonstrated significant enzyme inhibition. To validate and confirm these findings, it is crucial to examine the effects in vivo, as in vitro studies alone cannot fully replicate the complex interactions and metabolic processes occurring within a living organism. In vivo studies offer a deeper insight into the bioavailability, efficacy, and potential side effects of the extracts in a physiological context. Therefore, we proceeded to test the extracts’ inhibitory effects on α-amylase activity in vivo. After administering starch (2 g/kg), glycemia peaked at 2.18 ± 0.02 g/L at 60 min, as shown in Figure 5. It then gradually decreased to 1.79 ± 0.01 g/L at 90 min, 1.0 ± 0.005 g/L at 150 min, and stabilized around 0.94 ± 0.001 g/L. However, with the administration of AW (400 mg/kg), postprandial glycemia significantly decreased (*p* < 0.001 at 60 and 90 min, and *p* < 0.01 at 150 and 210 min), reaching 1.05 ± 0.06 g/L at 60 min, 0.89 ± 0.004 g/L at 90 min, 0.72 ± 0.005 g/L at 150 min, and 0.70 ± 0.001 g/L at 210 min relative to the control. Similarly, the presence of TG (400 mg/kg) significantly reduced postprandial glycemia (*p* < 0.001 at 60 and 90 min), resulting in values of 1.22 ± 0.008 g/L at 60 min and 1.21 ± 0.006 g/L at 90 min. Additionally, TM (400 mg/kg) significantly decreased postprandial glycemia (*p* < 0.001 at 60 and 90 min), with levels reaching 1.35 ± 0.008 g/L at 60 min and 1.05 ± 0.001 g/L at 90 min compared to the control. Acarbose administration (10 mg/kg) significantly reduced starch-induced hyperglycemia (*p* < 0.001) at 150 and 210 min, with values of 0.72 ± 0.005 g/L at 120 min and 0.57 ± 0.001 g/L at 210 min. After a starch overload, the area under the curve (AUC) in the control group was 81.29 ± 0.01 g/L/h. Pretreatment with AW (400 mg/kg) significantly inhibited the glycemic load (*p* < 0.001), resulting in an AUC of 50.03 ± 0.003 g/L/h. TG (400 mg/kg) inhibited the glycemic load (*p* < 0.05) with an AUC of 67.89 ± 0.001 g/L/h. TM (400 mg/kg) also showed significant inhibition (*p* < 0.01) with an AUC of 64.93 ± 0.003 g/L/h. Acarbose (10 mg/kg) demonstrated inhibitory activity (*p* < 0.05) with an AUC of 67.35 ± 0.006 g/L/h. These results indicate that AW, TG, and TM significantly inhibit α-amylase activity in vivo, resulting in lower post-meal blood glucose levels. The effectiveness of AW was particularly notable, showing the most substantial decrease in glycemia and glycemic load. This suggests that the increased concentration of total secondary metabolites in these extracts is vital for the inhibition of α-amylase, thereby controlling postprandial hyperglycemia effectively. Previous in vivo research indicated that *C. siliqua* extracts significantly inhibited α-amylase activity and improved blood glucose levels in type 2 diabetic rats. High-dose carob-treated (CS1000) and glibenclamide-treated (GD) groups had higher total protein and serum amylase levels compared to untreated diabetics (D0). The treated groups also had lower serum fasting blood glucose (FBG) and higher insulin levels. Additionally, the HOMA-B index improved significantly in the treated groups, indicating better beta-cell function. These results highlight the potential of *C. siliqua* extracts in managing diabetes [87].

#### 3.7.2. Intestinal α-Glucosidase

**Figure 6:**
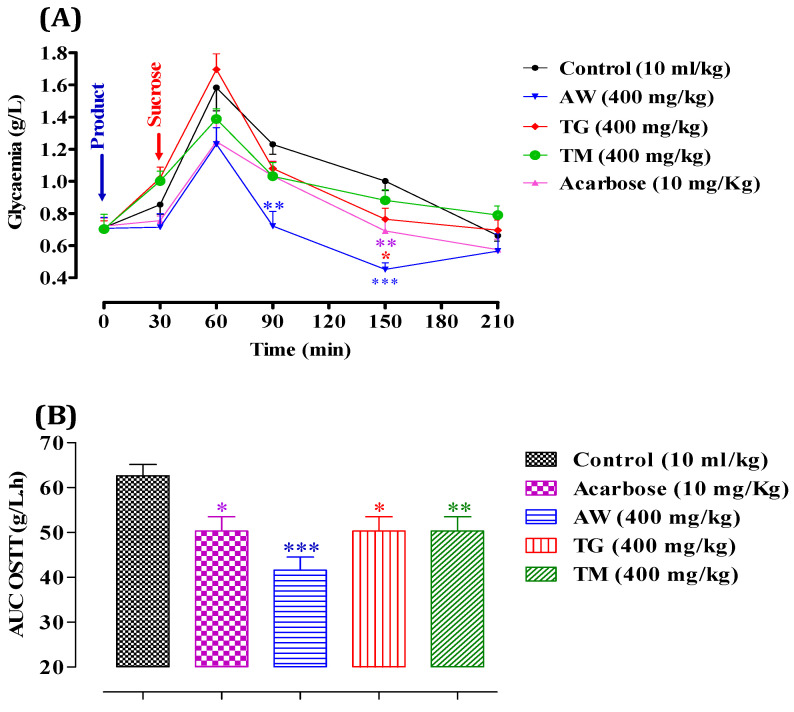
Impact of AW, TG, TM, and acarbose on glycemia following sucrose overload (2 g/kg) in normal rats (*n* = 6) (**A**). Area under the curve (AUC) for rats subjected to sucrose overload in the presence of AW, TG, TM, or acarbose (**B**). * *p* < 0.05; ** *p* < 0.01; *** *p* < 0.001. TG: Tizi-Ghnim site; TM: Timoulilt site; AW: Ait Waarda site.

We began by evaluating the inhibitory effects of ICPE on α-glucosidase in vitro, where we observed substantial enzyme inhibition. However, to validate these findings, in vivo studies are necessary. This step is crucial as in vitro experiments cannot fully replicate the intricate interactions and metabolic processes that occur within a living organism. Following sucrose administration (2 g/kg), glycemia peaked at 1.58 ± 0.003 g/L at 60 min (Figure 6), then gradually declined to 1.23 ± 0.001 g/L at 90 min, 1.0 ± 0.001 g/L at 150 min, and stabilized around 0.66 ± 0.01 g/L. However, with the administration of AW (400 mg/kg), postprandial glycemia significantly decreased (*p* < 0.01 at 90 min and *p* < 0.001 at 210 min), reaching 0.72 ± 0.001 g/L at 90 min and 0.45 ± 0.01 g/L at 210 min compared to the control. Similarly, TG (400 mg/kg) significantly reduced postprandial glycemia (*p* < 0.05 at 150 min), resulting in values of 0.76 ± 0.005 g/L at 150 min. Additionally, TM (400 mg/kg) and acarbose (10 mg/kg) did not exhibit significant inhibitory activity on α-glucosidase in vivo. After a sucrose overload, the area under the curve (AUC) in the control group was 62.61 ± 0.003 g/L/h. Pretreatment with AW (400 mg/kg) significantly reduced the glycemic load (*p* < 0.001), resulting in an AUC of 41.60 ± 0.001 g/L/h. TG (400 mg/kg) inhibited the glycemic load (*p* < 0.05) with an AUC of 50.32 ± 0.01 g/L/h, while TM (400 mg/kg) also showed significant inhibition (*p* < 0.01) with an AUC of 50.32 ± 0.001 g/L/h. Acarbose (10 mg/kg) demonstrated an inhibitory effect (*p* < 0.05) with an AUC of 50.32 ± 0.01 g/L/h. These findings suggest that AW, TG, and TM significantly inhibit α-glucosidase activity in vivo, leading to reduced postprandial blood glucose levels. The effectiveness of AW was particularly notable, showing the most substantial decrease in glycemia and glycemic load. This indicates that the greater amount of total secondary metabolites in these extracts are keys to inhibiting α-glucosidase, effectively controlling postprandial hyperglycemia. This study is pioneering in its exploration of the inhibitory activity of α-glucosidase in vivo using ICPE. While previous research has primarily focused on in vitro analyses, our work is the first to extend these investigations to a living organism, providing a more comprehensive understanding of the extract’s potential antidiabetic effects. Even though other studies have already demonstrated the antidiabetic effect of *C. siliqua* in vivo, they have done so using different methods [23].

This study offers a valuable understanding of the potential of using immature carob pulp at different stages of maturity (M1, M2, M3, M4, and M5) as a traditional alternative for treating diabetes. By demonstrating significant inhibitory effects on digestive enzymes and highlighting the stage with the highest polyphenol and flavonoid levels, along with antioxidant properties, our findings underscore the therapeutic promise of carob pulp. Given the gastrointestinal side effects associated with conventional treatments like acarbose, incorporating immature carob pulp could offer a beneficial, natural alternative for diabetes management.

### 3.8. Molecular Docking: Targeting α-Glucosidase and α-Amylase

**Figure 7:**
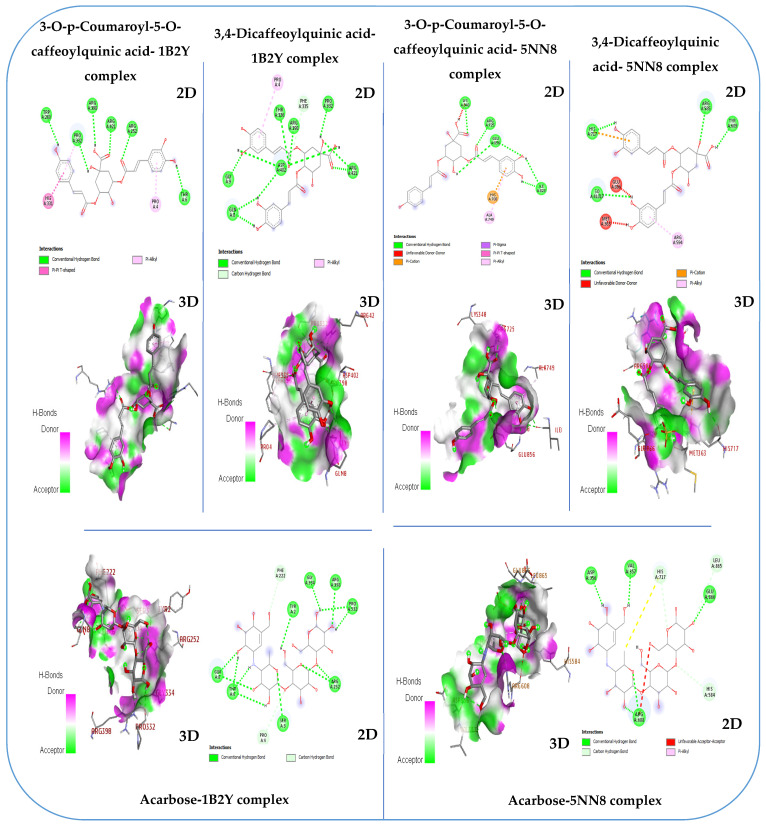
The 2D and 3D binding interactions of phytochemicals identified in the methanolic extract of unripe carob pods and acarbose (standard inhibitor) against the human α-glucosidase (PDB: 5NN8) and human α-amylase enzyme (PDB: 1B2Y).

Pancreatic α-amylase is a crucial enzyme in carbohydrate metabolism. It is also studied as a therapeutic target for diabetes treatment, as modulating its activity can help manage postprandial blood glucose levels. Therefore, α-amylase is important not only for digestion but also for its medical and therapeutic applications in diabetes management [88]. In this study, results obtained from molecular docking of ligands to human α-amylase (PDB: 1B2Y) revealed that the 3-O-p-Coumaroyl-5-O-caffeoylquinic acid molecule exhibited a higher binding affinity higher (−9.2 kcal/mol) for α-amylase than acarbose (−8.2 kcal/mol), which is a standard inhibitor of α-amylase. Additionally, quercetin 3-methyl ether exhibited a similar binding affinity to acarbose (−8.2 kcal/mol) (Table 7). The 3-O-p-coumaroyl-5-O-caffeoylquinic acid molecule stood out for its good affinity towards α-amylase, interacting effectively with several amino acid residues of the enzyme. Specifically, it demonstrated hydrogen bond interactions with Tyr 6, Arg 252, Trp 280, Pro 332, Arg 398, and Arg 421. Moreover, it exhibited a pi–alkyl interaction with Pro 4 and a pi–pi T-shaped interaction with His 331, offering deeper insight into its binding with α-amylase (Figure 7). α-glucosidase is a crucial enzyme in carbohydrate metabolism. It plays a crucial role in the digestive process by catalyzing the hydrolysis of α-glucose residues at the non-reducing ends of carbohydrates, such as starch and disaccharides. Due to its central role in carbohydrate digestion and absorption, α-glucosidase has become an important target in the treatment of diabetes. Inhibition of this enzyme can effectively delay glucose absorption, leading to a more gradual rise in blood sugar levels after meals [89]. In this study, the results obtained from molecular docking of ligands to human α-glucosidase (PDB:5NN8) revealed that the 3-O-p-coumaroyl-5-O-caffeoylquinic acid and 3,4-dicaffeoylquinic acid molecules exhibited higher binding affinities, with −8.2 kcal/mol and −7.9 kcal/mol (Table 7). The 3-O-p-coumaroyl-5-O-caffeoylquinic acid molecule stood out for its good affinity towards α-amylase, interacting effectively with several amino acid residues of the enzyme. Specifically, it demonstrated hydrogen bond interactions with Lys 348, Arg 725, Ile 823, and Glu 856, pi–cation interaction with His 708, and pi–alkyl interaction with Ala 749. Additionally, the 3,4-dicaffeoylquinic acid molecule interacted effectively with several amino acid residues of the enzyme. It demonstrated hydrogen bond interactions with Arg 585, Tyr 609, and His 717, thus providing further insight into its interaction with α-glucosidase (Figure 7). These findings indicate that the compounds in the methanolic extract of immature carob pods exhibit significant affinity for α-amylase and α-glucosidase enzymes. This affinity aligns with the inhibitory effects observed in our in vitro and in vivo studies, further supporting the potential of this extract as a natural inhibitor of enzymes involved in carbohydrate metabolism.

### 3.9. ADME Analysis

**Figure 8:**
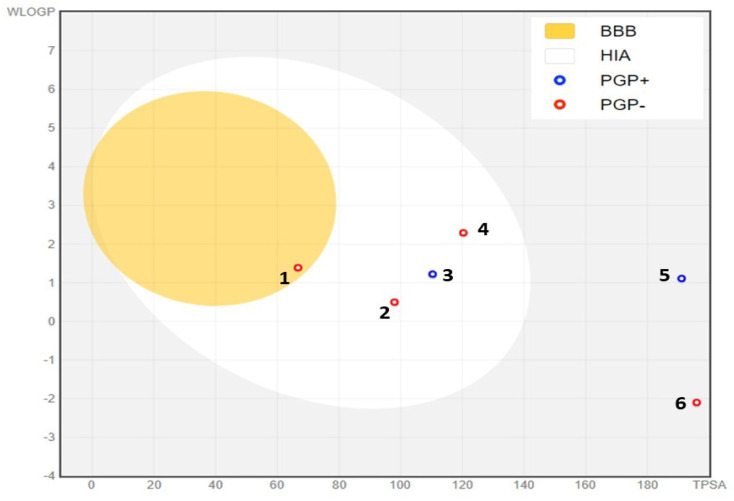
BOILED-Egg model of the GI absorption and BBB permeability of phytochemicals identified in methanolic extract of unripe carob pods. (1) Ferulic acid, (2) gallic acid, (3) catechin, (4) quercetin-3-methylether, (5) 3-O-p-coumaroyl-5-O-caffeoylquinic acid, (6) galloylquinic acid. PGP+: P-glycoprotein substrate; PGP−: non-substrate of P-glycoprotein.

In silico ADME studies are crucial for advancing pharmaceutical development, as they offer an affordable approach to forecasting a drug’s actions within the human body [90]. Leveraging computational models for early pharmacokinetic evaluation allows for the rapid identification of promising drug candidates, optimizing the development process. This approach reduces the risk of adverse side effects, lowers the probability of drug development failures, and enhances the likelihood of clinical success, making it a crucial asset in contemporary drug discovery and development [42]. To adhere to Lipinski’s rule of five and Veber’s rule, compounds that are considered appropriate for oral drug development should typically not violate more than one of the following criteria: (1) a maximum of 10 hydrogen bond acceptors (nitrogen or oxygen atoms), (2) an octanol–water partition coefficient log P (MLogP) of less than 5, (3) a molecular weight of less than 500 Daltons, and (4) no more than 5 hydrogen bond donors [91]. In our study, all compounds tested conformed to Lipinski’s guidelines, suggesting their potential for oral drug development, except 3-O-p-coumaroyl-5-O-caffeoylquinic acid and 3,4-dicaffeoylquinic acid. The blood–brain barrier (BBB) acts as a vital barrier between the systemic circulation and the central nervous system, safeguarding the brain through biochemical processes such as enzyme reactions and physical mechanisms like active efflux systems [92]. Our study found that only ferulic acid can cross the BBB, as indicated by the yellow region in Figure 8. Additionally, most of the compounds analyzed were identified as P-glycoprotein non-substrates (PGP-), except for 3-O-p-coumaroyl-5-O-caffeoylquinic acid and catechin, which were classified as PGP+ (Figure 8). A molecule that is efficiently absorbed by the intestine presents significant benefits in terms of bioavailability, efficacy, convenience, and tolerance, making it a strong candidate for oral drug development [93]. Notably, all the phytochemicals analyzed in our study exhibit high intestinal absorption, except galloylquinic acid, 3-O-p-coumaroyl-5-O-caffeoylquinic acid, and 3,4-dicaffeoylquinic acid. Cytochrome P450, a key enzyme for detoxification, is primarily found in the liver [94]. Our analysis showed that most compounds are neither inhibitors nor substrates of CYP450 enzymes, particularly CYP1A2, except for quercetin-3-methyl ether, which was identified as a CYP1A2 inhibitor (Table 8). This suggests a lower likelihood of interference with drug metabolism, enhancing the safety profile of the phytochemicals.

## 4. Conclusions

Maturity can be used to predict the potential content of secondary metabolites with antioxidant capacity, such as polyphenols and flavonoids. The phytochemical composition and antioxidant capacity of immature carob pulp exhibited variation concerning the maturity stage, as evidenced by this study. The stage containing the highest amount of polyphenols demonstrated a notable antidiabetic effect, suggesting that these compounds may play a role in the prevention and management of diabetes. It can be posited that this activity is responsible for the presence of a significant amount of 3-O-p-coumaryol-5-O-caffeoylquinique in the carob pulp extracts. In light of these new findings, further rigorous studies must be conducted on how this compound functions as a medicinal agent in a variety of animal models to gain a comprehensive understanding of its mechanism of action concerning blood sugar control. Our study’s findings indicate that immature carob pulp extracts contain active principles. The in silico analysis reveals that these compounds exhibit varied affinities with key enzymes involved in carbohydrate metabolism and also possess favorable properties for the development of oral medications. In particular, immature carob pulp extracts offer a valuable opportunity for the development of new natural therapeutic strategies against diabetes. Their potential as effective alternatives to established synthetic inhibitors underscores the importance of continuing research to explore and leverage these compounds in clinical applications.

## Figures and Tables

**Table 1:** Change in total sugar content of unripe carob pulp during ripening stages.

	Total Sugar mg GE/100 g FM		
	TG	TM	AW
M1	1720 ± 76 ^dA^	1709 ± 73 ^cA^	1889 ± 125 ^bA^
M2	1814 ± 84 ^cdA^	1721 ± 92 ^bcA^	1636 ± 37 ^cA^
M3	1880 ± 7 ^bcB^	1987 ± 72 ^aA^	1982 ± 55 ^abA^
M4	2032 ± 22 ^bA^	1880 ± 25 ^abA^	1940 ± 28 ^abA^
M5	2134 ± 56 ^aA^	1904 ± 21 ^aA^	2098 ± 34 ^aA^

The results are shown as the mean value ± SD (n = 3). Distinct lowercase letters indicate statistically significant variations (*p* < 0.05) across unripening phases, while distinct capital letters indicate statistically significant variations (*p* < 0.05) between provenances as determined by the Tukey test. TG: Tizi-Ghnim site; TM: Timoulilt site; AW: Ait Waarda site; (M1, M2, M3, M4, and M5): stages of the immature phase of carob pulp; GE: glucose equivalent; FM: fresh matter.

**Table 2:** Change in total phenolic content, total flavonoid content, and total condensed tannins of unripe carob pulp during ripening stages.

	TPC mg GAE/100 g FM			TFC mg QE/100 g FM			TCT mg CE/100 g FM		
	TG	TN	AW	TG	TN	AW	TG	TN	AW
M1	2813 ± 58 ^bA^	3014 ± 59 ^bA^	2734 ± 99 ^aA^	826.2 ± 41 ^bB^	298.7 ± 20 ^cC^	1423 ± 78 ^aA^	613.9 ± 74 ^bA^	231.2 ± 70 ^cB^	567.9 ± 21 ^bA^
M2	3475 ± 189 ^aB^	3819 ± 226 ^aA^	2765 ± 144 ^aC^	925.4 ± 13 ^aAB^	1034 ± 57 ^aA^	830.1 ± 87 ^bB^	848.1 ± 19 ^aB^	1472 ± 28 ^aA^	756.1 ± 92 ^aC^
M3	2750 ± 98 ^bcA^	2368 ± 43 ^cB^	2209 ± 87 ^bC^	900.6 ± 65 ^abA^	827.5 ± 49 ^bA^	848.4 ± 52 ^bA^	628.5 ± 93 ^bA^	633.7 ± 61 ^bA^	539.6 ± 35 ^bB^
M4	2532 ± 48 ^cA^	2120 ± 151 ^cB^	1947 ± 55 ^cB^	173.2 ± 11 ^dA^	196.2 ± 11 ^dA^	161.2 ± 13 ^cA^	724.7 ± 22 ^abA^	706.9 ± 37 ^bAB^	565.8 ± 18 ^bB^
M5	2744 ± 39 ^bcA^	2391 ± 76 ^cC^	2595 ± 91 ^aB^	294 ± 12 ^cA^	249.6 ± 16 ^cdA^	305.6 ± 12 ^cA^	75.7 ± 5.5 ^cA^	71.4 ± 3.3 ^dA^	77.1 ± 6.1 ^cA^

The results are shown as the mean value ± SD (n = 3). Distinct lowercase letters indicate statistically significant variations (*p* < 0.05) across unripening phases, while distinct capital letters indicate statistically significant variations (*p* < 0.05) between provenances as determined by Tukey test. TG: Tizi-Ghnim site; TM: Timoulilt site; AW: Ait Waarda site; (M1, M2, M3, M4, and M5): stages of the immature phase of carob pulp; GAE: gallic acid equivalent; QE: quercetin equivalent; CE: catechin equivalent; FM: fresh matter.

**Table 3:** HPLC-UV MS/MS phenolic profile of the methanolic extracts of immature carob pulp (MEICP) from various locations (TG, TM, and AW).

Compounds	Chemical Formula	RT (min)	% Area			Ref
			TM	AW	TG	
3-O-p-coumaroyl-5-O-caffeoylquinic acid	C_25_H_24_O_11_	4.28	53.40	63.62	55.84	N
Quercetin 3-methyl ether	C_16_H_12_O_7_	5.26	13.87	12.91	13.83	N
Gallic acid	C_7_H_6_O_5_	6.60	11.24	5.17	2.35	S
3,4-Dicaffeoylquinic acid	C_25_H_24_O_12_	8.43	3.04	2.85	5.06	N
Galloylquinic acid	C_14_H_16_O_10_	10.95	12.55	n.d.	n.d.	(1)
Ferulic acid	C_10_H_10_O_4_	13.52	0.34	0.37	0.60	S
Catechine	C_15_H_14_O_6_	17.84	n.d.	0.37	n.d.	S

RT: retention time; n.d.: not detected; ref: reference; N: Nist/MSMS; S: standard; MEICP: methanolic extracts of immature carob pulp; TG: Tizi-Ghnim site; TM: Timoulilt site; AW: Ait Waarda site; (1): [39].

**Table 4:** A comprehensive overview of additional pre-clinical or behavioral signs of acute toxicity.

	Doses	2 g/kg	1 g/kg	0.5 g/kg
Symptoms				
Locomotion and mobility		Normal	Normal	Normal
Hair loss or erection		-	-	-
Diarrhea		-	-	-
Abnormal agitation		-	-	-
Anorexia		-	-	-
Spontaneous startle		-	-	-
Isolation in the corner		-	-	-
Mortality		-	-	-
Body weight gain		Normal	Normal	Normal

-: the absence or non-occurrence of the sign.

**Table 5:** Inhibition of α-amylase activity by TG, TM, AW, and acarbose.

Extraits	IC_50_ µg/mL
TG	0.591 ± 0.104 a
TM	0.450 ± 0.239 ab
AW	0.405 ± 0.247 ab
Acarbose (control)	0.098 ± 0.004 b

Values are presented as mean ± SEM (n = 3). Data were analyzed using one-way ANOVA with post-hoc Tukey’s test at a 5% significance level. Means within the same column that have different letters are significantly different (*p* < 0.05). TG: Tizi-Ghnim site; TM: Timoulilt site; AW: Ait Waarda site.

**Table 6:** Inhibitory activity of α-glucosidase by TG, TM, AW, and acarbose.

Extraits	IC_50_ µg/mL
TG	0.063 ± 0.002 ^b^
TM	0.086 ± 0.014 ^a^
AW	0.065 ± 0.004 ^b^
Acarbose (control)	0.089 ± 0.004 ^a^

Values are presented as mean ± SEM (n = 3). Statistical analysis was performed using one-way ANOVA with Tukey’s post-hoc test at a 5% significance level. Means within the same column marked with different letters are significantly different (*p* < 0.05). TG: Tizi-Ghnim site; TM: Timoulilt site; AW: Ait Waarda site.

**Table 7:** Summary of molecular docking studies on phytochemicals identified in the methanolic extract of unripe carob pods, targeting human α-glucosidase and α-amylase.

	α-Amylase (PDB: 1B2Y)		α-Glucosidase (PDB: 5NN8)	
Compound Name	Affinity (kcal/mol)	Interaction Site	Affinity (kcal/mol)	Interaction Site
Acarbose (standard inhibitor)	−8.2	Tyr 2, Ser 3, Pro 4, Thr 6, Gln 8, Phe 222, Arg 252, Pro 332, Gly 334, Arg 398.	−7.2	Asp 356, Val 357, Arg 608, His 584, His 717, Leu 865, Glu 866.
3-O-p-coumaroyl-5-O-caffeoylquinic acid	−9.2	Pro 4, Tyr 6, Arg 252, Trp 280, His 331, Pro 332, Arg 398, Arg 421	−8.2	Lys 348, His 708, Arg 725, Ala 749, Ile 823 Glu 856
Quercetin 3-methyl ether	−8.2	Arg 267, Arg 303, Thr 314, Arg 346, Asp 356	−7.1	Met 363, Arg 585, Arg 594
Gallic acid	−5.9	Val 129, Gly 181, Tyr 182	−5.8	His 708, Gln 715, Glu 748, Ala 749, Ile 823, Glu 856.
3,4-dicaffeoylquinic acid	−8.2	Pro4, Gln 8, Gly 9, Pro 332, Phe 335, Thy 336, Arg 398, Asp 402, Arg 421	−7.9	Met 363, Arg 585, Arg 594, Tyr 609, His 717, Glu 866,
Galloylquinic acid	−7.2	Arg 10, Arg 252, Ser 289, Gly 334, Asp 402, Gly 403	−6.4	Leu 195, Arg 585, Tyr 609
Ferulic acid	−6.3	Arg 552, Ser 289, Pro 332, Phe 335, Arg 424	−6.6	His 708, Glu 721, Arg 725, Tyr 822, Gly 855, Glu 856
Catechin	−7.8	Asn 301, Ile 312, Thr 314, Asp 317, Arg 346, Asn 352	−6.8	Pro 194, Leu 195, Phe 490, Thr 491, Leu 577, Ile 581, Tyr 609

**Table 8:** Evaluation of the pharmacokinetic properties (ADME) of phytochemicals identified in methanolic extract of unripe carob pods (in silico).

	Physicochemical Properties					Lipophilicity		Druglikeness		Pharmacokinetics		
Compounds	MW g/mol	HBA	HBD	TPSA Å²	ROTB	MlogP	WlogP	Lipinski’s	Verber’s	GI Absorption	BBB Permeation	CYP1A2 Inhibitor
3-O-p-coumaroyl-5-O-caffeoylquinic acid	500.4	11	6	191.0	9	0.1	1.1	3	1	Low	No	No
Quercetin 3-methyl ether	316.2	7	4	120.3	2	−0.3	2.2	0	0	High	No	No
Gallic acid	170.1	5	4	97.9	1	−0.1	0.5	0	0	High	No	No
3,4-dicaffeoylquinic acid	516.4	12	7	211.2	9	−0.3	0.8	3	1	Low	No	No
Galloylquinic acid	344.2	10	8	195.8	3	−2.7	−2.1	1	1	Low	No	No
Ferulic acid	194.1	4	2	66.7	3	1.0	1.3	0	0	High	Yes	No
Catechin	290.2	6	5	110.3	1	0.2	1.2	0	0	High	No	No

MW: molecular weight; ROTB: rotatable bonds; HBD: hydrogen bond donor; HBA: hydrogen bond acceptors; WLogP: lipophilicity; TPSA: topological polar surface area; MLogP: octanol–water partition coefficient; GI: gastrointestinal; BBB: blood–brain barrier.

## Data Availability

All the data relevant to this study have been added within the manuscript.
